# Ciliary Beating Recovery in Deficient Human Airway Epithelial Cells after Lentivirus *Ex Vivo* Gene Therapy

**DOI:** 10.1371/journal.pgen.1000422

**Published:** 2009-03-20

**Authors:** Brigitte Chhin, Didier Negre, Olivier Merrot, Jacqueline Pham, Yves Tourneur, Denis Ressnikoff, Martine Jaspers, Mark Jorissen, François-Loïc Cosset, Patrice Bouvagnet

**Affiliations:** 1 Université de Lyon, Lyon, France; 2 Université de Lyon (UCB-Lyon1), IFR128, Lyon, France; 3 INSERM, U758, Lyon, France; 4 Ecole Normale Supérieure de Lyon, Lyon, France; 5 Hospices Civils de Lyon, Hôpital de la Croix-Rousse, Service ORL, Lyon, France; 6 INSERM, UMR886, Cardioprotection, Lyon, France; 7 Centre Commun de Quantimétrie, Université de Lyon, Lyon, France; 8 Ear, Nose, and Throat Department, Head and Neck Surgery, Leuven, Belgium; 9 Hospices Civils de Lyon, Groupe Hospitalier Est, Laboratoire Cardiogénétique, Bron, France; 10 INSERM, CIC 201, Hôpital Louis Pradel, Bron, France; Johns Hopkins University School of Medicine, United States of America

## Abstract

Primary Ciliary Dyskinesia is a heterogeneous genetic disease that is characterized by cilia dysfunction of the epithelial cells lining the respiratory tracts, resulting in recurrent respiratory tract infections. Despite lifelong physiological therapy and antibiotics, the lungs of affected patients are progressively destroyed, leading to respiratory insufficiency. Recessive mutations in *Dynein Axonemal Intermediate chain type 1* (*DNAI1*) gene have been described in 10% of cases of Primary Ciliary Dyskinesia. Our goal was to restore normal ciliary beating in DNAI1–deficient human airway epithelial cells. A lentiviral vector based on Simian Immunodeficiency Virus pseudotyped with Vesicular Stomatitis Virus Glycoprotein was used to transduce cultured human airway epithelial cells with a cDNA of *DNAI1* driven by the Elongation Factor 1 promoter. Transcription and translation of the transduced gene were tested by RT–PCR and western blot, respectively. Human airway epithelial cells that were DNAI1–deficient due to compound heterozygous mutations, and consequently had immotile cilia and no outer dynein arm, were transduced by the lentivirus. Cilia beating was recorded and electron microscopy of the cilia was performed. Transcription and translation of the transduced *DNAI1* gene were detected in human cells treated with the lentivirus. In addition, immotile cilia recovered a normal beat and outer dynein arms reappeared. We demonstrated that it is possible to obtain a normalization of ciliary beat frequency of deficient human airway epithelial cells by using a lentivirus to transduce cells with the therapeutic gene. This preliminary step constitutes a conceptual proof that is indispensable in the perspective of Primary Ciliary Dyskinesia's *in vivo* gene therapy. This is the first time that recovery of cilia beating is demonstrated in this disease.

## Author Summary

This manuscript reports on a successful gene therapy attempt on human airway epithelial cells of a patient suffering from Primary Ciliary Dyskinesia. In this autosomal recessive disease, cilia of the epithelial cells that border the upper and lower respiratory tracks are not functioning. As a result, patients suffer from recurrent airway infections leading progressively to respiratory insufficiency. There is no treatment as of today that could restore normal ciliary beating. In this report, we showed that it is feasible to transfer a therapeutic gene to human airway epithelial cells with a lentivirus. This transferred gene is transcribed and expressed. Moreover, defective cells that had immotile cilia due to compound heterozygous mutations in the *DNAI1* gene recovered ciliary beating after treatment with a lentivirus containing a normal *DNAI1* gene. This is the first report on gene therapy in Primary Ciliary Dyskinesia. Since lentivirus is able to insert therapeutic genes into the cell genome, this result may have impact on *in vivo* gene therapy in this disease and in diseases related to human epithelial airway cells such as cystic fibrosis.

## Introduction

Primary Ciliary Dyskinesia (PCD, OMIM #242650) is an inherited disease mainly characterized by dysfunction of airways' motile cilia. The prevalence is approximately 1 in 12,000–20,000 [1]–[3]. About 50% of patients affected by PCD have a *situs inversus* which results from monocilia dysfunction at the embryonic node [4]. This association is referred to as Kartagener's syndrome (OMIM #244400) [5]. PCD causes chronic sinus and bronchial respiratory infections that begin early in life, leading to nasal polyps and bronchiectasis. Males are frequently sterile due to dysfunctional spermatozoa flagella [6]. Other symptoms can also be associated with PCD like hydrocephalus, anosmia, retinitis pigmentosa and congenital heart diseases [7]–[10].

The disorder is genetically heterogeneous and in most cases, inheritance is autosomal recessive but X-linked inheritance patterns were also described [11]. Several loci and some genes have been identified, as *DNAI1* (Ensembl ENSG00000122735), *DNAH5* (ENSG00000039139), *DNAH11* (ENSG00000105877), *RPGR* (ENSG00000156313), *TXNDC3* (ENSG00000086288), *OFD1* (ENSG00000046651), *DNAI2* (ENSG00000171595) and *KTU* (alias *C14orf104*) (OTTHUMG00000152331) genes [10], [12]–[19]. The first gene described to be responsible for PCD and Kartagener syndrome was *DNAI1* gene [18],[20]. Eighteen mutations in *DNAI1* gene were reported, and Zariwala *et al.* evidenced a founder effect for the most frequent mutation (c.48+2_48+3insT) [21]. Moreover, the authors estimated that mutations in *DNAI1* gene represent about 10% of PCD cases.

*DNAI1* encodes an axonemal dynein intermediate chain, a component of the outer dynein arm (ODA). Dyneins are molecular motors which produce energy for microtubules doublets sliding in the axoneme. To date, no etiologic treatment of PCD is available and on the long range, PCD leads to respiratory insufficiency and lung transplant.

We hypothesized that gene therapy could restore ciliary function in *DNAI1*-mutated airway epithelial cells to prevent patients from infectious complications. To introduce genetic material into cells we focused on lentiviral gene transfer because lentivirus has the property to integrate its genetic material into host cell genome even in non-replicating cell [22]. Moreover, lentivirus is weakly immunogenic unlike recombinant adenovirus which efficiency was reported to decrease after several administrations in a clinical study of patients suffering from cystic fibrosis [23]. Lentiviral-derived vectors used in gene therapy were principally based on SIV (simian immunodeficiency virus) or HIV (human immunodeficiency virus). For this latter one, gene transfer efficiency into bronchial epithelium in mice was already demonstrated [24].

We decided to modify a SIV-based vector, previously described by Negre *et al*. to efficiently transduce mature human dendritic cells [25],[26], and to transduce human airway epithelial cells (HAECs) cultured as described by Jorissen *et al*. [27]. First, we showed here that normal HAECs were efficiently transduced by SIV-based vector containing *eGFP* gene. Then, we validated lentiviral vectors' constructions containing *DNAI1* cDNA sequence and showed that transduced *DNAI1* is transcribed and expressed. Finally, we demonstrated that transduction of *DNAI1*-mutated HAECs with wild-type *DNAI1* can restore ciliary beating and that ODA are binding again to microtubules.

## Results

### Transduction of HAECs with pGFP

**Figure 1:**
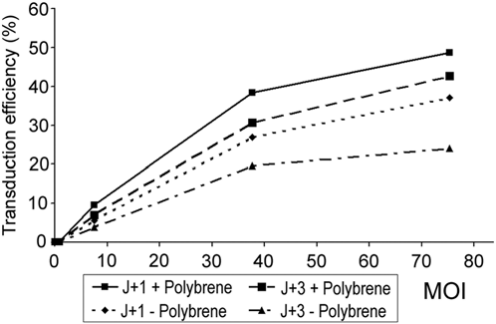
Transduction efficiency analysis by FACS two days after HAECs infection with pGFP vector. MOI, multiplicity of infection (3 values of MOI were tested 7, 35, and 75); J+1, one day after biopsy, ciliated cells in suspension; J+3, three days after biopsy, de-differentiated and adherent cells; +, with use of Polybrene; −, without use of Polybrene. These results were obtained from a single experiment in each condition. MOI is the ratio of infectious agents (e. g. lentivirus) to infection targets (e. g. cells).

To estimate whether HAECs could be transduced by a SIV-based lentivirus pseudotyped with VSV-G, normal HAECs were infected with pGFP a vector containing *eGFP* as a reporter gene in a variety of conditions [25],[26]. Three parameters were investigated: (1) the multiplicity of infection (MOI), (2) the moment of infection and (3) whether using a polycation, Polybrene, or not. Two different moments of infection during Jorissen's culture were tested: at J+1, cells were ciliated and in suspension or at J+3, cells were de-differentiated and adherent. Two days post-infection, reporter gene expression was analyzed by FACS (Figure 1). In any of the selected conditions, HAECs were transduced but the proportion of transduced cells seemed to be dependant on MOI irrespective of the other parameters, and apparently higher at MOI 75. Then, transduction of cells infected at J+1 seemed more efficient compared to cells infected at J+3. At MOI 75, approximately 38% of cells infected at J+1 were transduced versus 20% for cells infected at J+3. Moreover, these results seemed to be improved by the use of Polybrene. Finally, at MOI 75, transduction efficiency was quantified at about 38% for cells infected at J+1 without Polybrene compared to approximately 50% for cells infected at J+1 with the use of Polybrene. These experiments which were not repeated, demonstrated that HAECs could be transduced in a variety of conditions and that J+1 with Polybrene at a MOI of 75 were presumably the best experimental conditions for HAECs infection. Therefore, we selected these conditions for further experiments.

### *DNAI1* cDNA Cloning in Lentiviral Vectors

**Figure 2:**
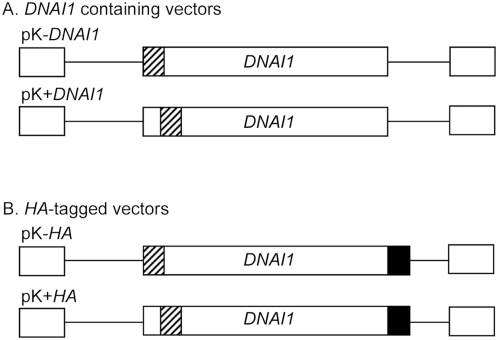
Lentiviral vectors containing *DNAI1* cDNA sequence with or without *HA* tag. (A) pK-*DNAI1* and pK+*DNAI1* were derived from pGFP, with *DNAI1* cDNA sequence in place of *eGFP* gene. pK-*DNAI1* contains an intact *DNAI1* cDNA sequence associated with a modified Kozak sequence (atccccaccauga), whereas pK+*DNAI1* contains an intact Kozak sequence associated with a modified *DNAI1* cDNA sequence (c.4A>G; p.I2V). (B) *HA* tag addition at 3′ side of *DNAI1* cDNA sequence in pK-*DNAI1* and pK+*DNAI1* vectors forms pK-*HA* and pK+*HA* vectors, respectively. Striped box: site of sequence modification; filled box: *HA* tag sequence. Empty boxes located 5′ and 3′ to cDNA sequence represent 5′ and 3′ LTR (Long Terminal Repeat), respectively.

Full-length *DNAI1* cDNA was cloned into the lentiviral vector in place of *eGFP* gene. Two constructions were obtained using *Bam*H I and *Xho* I restriction sites (pK-*DNAI1*) or *Nco* I and *Xho* I (pK+*DNAI1*), and resulted respectively in an intact *DNAI1* cDNA associated with a modified Kozak sequence which could prevent normal translation, or an intact Kozak sequence associated with a modified *DNAI1* cDNA which could result in a dysfunctional protein (Figure 2A). Then, to differentiate endogene from exogene *DNAI1*, a hemagglutinin tag (*HA*) was added at the 3′ side of *DNAI1* cDNA sequence in each plasmid which resulted in two additional vectors: pK-*HA* or pK+*HA* (Figure 2B).

### Detection of *DNAI1* Gene Expression in Transduced Normal HAECs

**Figure 3:**
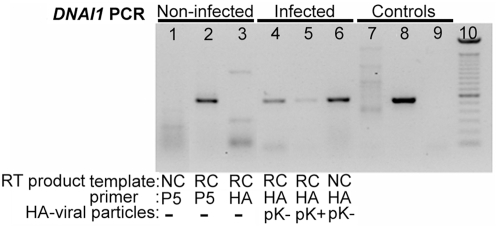
*HA*-tagged *DNAI1* gene transcription analysis by RT–PCR, in transduced normal HAECs. RT products were obtained using non-ciliated (NC) or re-ciliated (RC) HAECs mRNA and *DNAI1* (P5) or *HA*-tag (*HA*) specific primers. All PCR products were obtained using *DNAI1* cDNA specific primers. (1–3) Non-infected cells: (1) NC HAECs and P5 primer, (2) RC HAECs and P5 primer, (3) RC HAECs and *HA* primer; (4–6) Infected cells with *HA*-tagged *DNAI1* and RT with *HA* primer: RC HAECs infected with (4) pK-*HA* or (5) pK+*HA*, (6) NC HAECs infected with pK-*HA*; (7–9) PCR control templates (no RT) were (7) genomic DNA, (8) pK-*HA* vector, (9) no DNA; (10) 100 bp DNA ladder. The symbol “-” means no infection by viral particles.

Normal HAECs were transduced at J+1 with Polybrene at a MOI of 75 since these conditions gave satisfactory results as evaluated with *eGFP*. First, mRNA extracts from transduced HAECs were controlled by PCR with *alpha-tubulin* specific primers for absence of genomic DNA (gDNA) contamination (not shown). Second, we confirmed that non-ciliated HAECs (NC) do not express *DNAI1* because *DNAI1* specific RT primer (P5) led to an absence of amplification by contrast to re-ciliated HAECs (RC) template (Figure 3, lanes 1 and 2). Third, *DNAI1* mRNA was not amplified using a *HA*-tag RT specific primer (*HA*) from non-infected re-ciliated HAECs (Figure 3, lane 3). To test *DNAI1* transcription from lentiviral vectors, *HA*-tagged *DNAI1* gene transcription was revealed by RT-PCR using *HA-DNAI1* specific primers. Re-ciliated HAECs infected by particles containing *HA*-tagged *DNAI1* with either an exact Kozak sequence (pK+*HA*) or a modified Kozak sequence (pK-*HA*) both transcribed *HA*-tagged *DNAI1* (Figure 3, lanes 4 and 5). Non-ciliated HAECs infected by pK-*HA* particles also expressed *HA*-tagged *DNAI1* (Figure 3, lane 6). In conclusion, *DNAI1* transcription is efficient from vectors containing a conserved or a modified Kozak sequence and transcription of the transduced *DNAI1* cDNA driven by Elongation Factor-1 promoter is active in non-ciliated and re-ciliated HAECs.

### Detection of DNAI1 Proteins in Transfected 293Bosc Cells

To detect DNAI1 protein translation from lentiviral vectors, 293Bosc cell line which naturally does not express *DNAI1* was transfected by each lentiviral vector using ExGen500.

**Figure 4:**
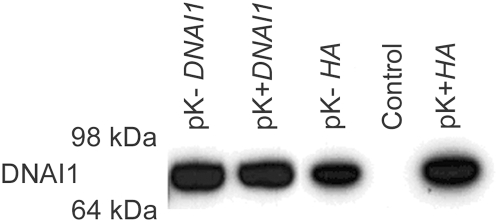
DNAI1 protein detection with the use of DNAI1 anti-dyn69 antibody. Western blot analysis of proteins lysates from transfected 293Bosc cells with (1) pK-*DNAI1*, (2) pK+*DNAI1*, (3) pK-*HA*, (5) pK+*HA*, vectors; (4) control of transfection.

DNAI1 protein was detected by Western blot with specific anti-DNAI1 antibodies after 293Bosc cells' transfection with the Kozak modified (pK-*DNAI1*) or Kozak conserved (pK+*DNAI1*) *DNAI1* sequence (Figure 4, lane 1 and 2). DNAI1 protein was also detected after 293Bosc cells' transfection with the vectors containing *HA* appended to the 3′ end of *DNAI1* sequence (Figure 4, lane 3: modified Kozak and lane 5: conserved Kozak sequence). By contrast, no DNAI1 protein could be detected by Western blot in protein lysate of non-transfected 293Bosc cells with anti-DNAI1 antibodies (Figure 4, lane 4).

Moreover, immunofluorescence assays showed that in pK-*HA* transfected 293Bosc cells, cytoplasmic *HA*-tagged DNAI1 proteins could be specifically detected by HA antibodies (Figure S1).

### Transduction of *DNAI1–*Mutated HAECs

In the second set of experiments, transduction was performed on *DNAI1*-mutated HAEC at J+1 and cells were cultured to generate ciliated vesicles. *DNAI1*-mutated HAECs were transduced with pK-*DNAI1* or pK-*HA* to determine if immotile cilia might recover a beat. The same protocol of transduction was used as for normal HAECs. We confirmed *DNAI1*-mutated HAECs transduction efficiency with pGFP vector, as de-differentiated cells at J+3 and re-ciliated cells at J'+17 (17 days post-collagen digestion) expressed GFP protein (Figure S2).

**Figure 5:**
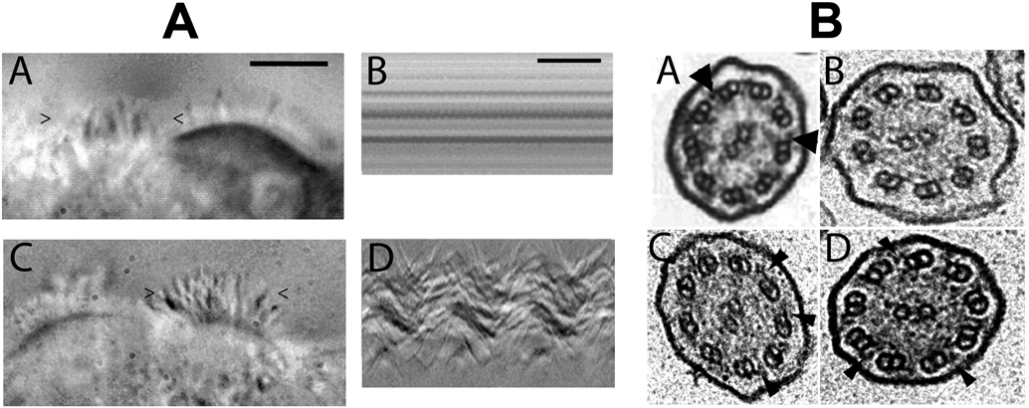
Images of transduced *DNAI1*–mutated HAECs and electron microscopic sections of cilia. (A) At J'+31, vesicles had cells with cilia transduced with either pGFP (A-A) or pK-*DNAI1* vectors (A-C). However, cilia of cells transduced with pGFP were immotile whereas cells transduced with pK-*DNAI1* vectors were beating. Figures (A-B) and (A-D) represent the variations of the video signal during the time of recording (400 msec) along a virtual line delimited by the “>” and “<” signs on figures (A-A) and (A-C), respectively. The periodic beat of cilia is clearly visible on figure (A-D) compared to the immotility of cilia on figure (A-B). Black bar on figure (A-A): 10 µm. Black bar on figure (A-D): 100 msec. *DNAI1–mutated* HAECs transduced with pK-*HA* vector containing *HA*-tagged *DNAI1* cDNA sequence gave identical results (data not shown). See Video S1 and Video S2. (B) Axoneme ultrastructural analysis by TEM (A-D): (B-A) Normal HAEC. (B-B) *DNAI1–mutated* HAEC treated with pGFP. (B-C) *DNAI1–mutated* HAEC treated with pK-*DNAI1* vector. (B-D) *DNAI1–mutated* HAEC treated with pK-*HA* vector. Arrowheads indicate ODA. Axoneme diameter is about 0.2 µm.

After re-differentiation, GFP transduced HAECs were covered with cilia (Figure 5A-A) but these cilia were immotile. The variation of optic signal along a line crossing the cilia during 400 msec does not show any movement (Figure 5A-B). This immotily is also visible on video recording (Video S1). By contrast, ciliary beating was recorded on *DNAI1*-mutated HAECs transduced with either pK-*DNAI1* or pK-*HA* vector (Figure 5A-C). This beat is demonstrated by recording the variation of optic signal along a line crossing cilia. Waves are clearly visible (Figure 5A-D) that evidenced the periodic beat of cilia. This active beat is also visible on video recording (Video S2). At J'+30, a ciliary beat frequency (CBF) was measured for pK-*DNAI1* and pK-*HA* transduced cells. CBF of HAECs with beating cilia were 9.95±1.23 Hz and 11.31±0.85 Hz, for pK-*DNAI1* and pK-*HA* treated cells, respectively. These values fall into the range of control HAECs (from 7 to 11 Hz). Cilia length in *DNAI1*-mutated HAECs was estimated at 6 µm in all cases and did not depend on *DNAI1* treatment and cilia beating.

**Figure 6:**
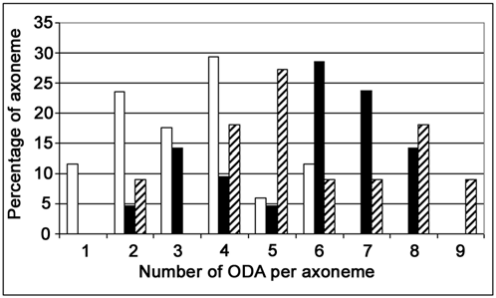
Distribution of the number of ODA per axoneme in *DNAI1–mutated* infected HAECs. X axis, number of ODA per axoneme; Y axis, percentage of axonemes with a given number of ODA. Empty bars, *DNAI1–mutated* HAECs infected with pGFP; filled bars, cells infected with pK–*DNAI1*; striped bars, cells infected with pK-*HA*.

In *DNAI1*-mutated HAECs' axoneme, ultrastructure analysis by TEM showed that ODA were absent or shorter than in normal HAECs (Figure 5B-B). TEM on axonemes of *DNAI1*-treated cells were analyzed and some cilia had normal amounts of ODA, while in other cilia the ODA were partially absent (Figure 5B-C and 5B-D). The average number of ODA per axoneme was 3.29±1.53 in pGFP infected *DNAI1*-mutated HAECs. ODA increased significantly to 5.67±1.83 (p<0.0001) and 5.73±2.10 (p<0.002) in *DNAI1*-mutated HAECs treated with pK-*DNAI1* and pK-*HA*, respectively. By contrast, there was no significant difference between *DNAI1*-mutated HAECs treated by pK-*DNAI1* or pK-*HA*. The distribution of the number of ODA per axoneme presented a single peak in pGFP infected *DNAI1*-mutated HAECs but two peaks in pK-*DNAI1* and pK-*HA* treated cells (Figure 6). IDA analysis showed no difference between control and *DNAI1*-treated cells (data not shown).

## Discussion

In the present study, we demonstrated that a SIV-based vector pseudotyped with VSV-G protein, previously described to efficiently transduce human dendritic cells [25],[26], could also efficiently infect normal cultured HAECs [27]. Previous studies showed that Murine Leukemia virus (MuLV) [28] and Feline Immunodeficiency Virus pseudotyped with VSV-G envelope [29] could transduce HAECs in culture. Though, this transduction was only possible from the basolateral surface of polarized HAECs. We did not know whether a Simian Immunodeficiency Virus would transduce although we could foresee that transduction would be more efficient from the basolateral surface of HAECs since it was pseudotyped with VSV-G. We did not know also whether de-differentiated cells would be transduced. It appeared that whatever the conditions used in this study, a certain proportion of cultured HAECs were infected. We then selected the conditions that seemed to provide the higher percentage of infected cells in culture. In any case, these conditions are strikingly different from *in vivo* situation where HAECs are a component of a complex epithelium recovered by a thin layer of mucus. Moreover, Polybrene which seemed to improve viral infection in cultured cells cannot be used *in vivo* due to side-effects. In a next step, we demonstrated transcription and translation of the transduced *DNAI1* cDNA. Finally, we showed that *DNAI1*-mutated HAECs treated by *DNAI1* gene transfer recovered ciliary beating whereas GFP-treated cells' cilia remained immotile, thus supporting our concept that PCD gene therapy was possible using *DNAI1* gene. This conceptual proof is essential in the perspective of a human gene therapy.

Since it was impossible to obtain a vector containing a conserved Kozak sequence associated with a normal *DNAI1* cDNA sequence, we decided to construct two vectors: one with a modified Kozak sequence upstream a conserved *DNAI1* cDNA sequence (pK-*DNAI1*), and another one with a conserved Kozak sequence but associated with a modified *DNAI1* cDNA sequence (pK+*DNAI1*). However, both vectors turned out to be equally efficient in terms of transcription and translation. Moreover, the addition of a *HA* tag at the 3′ end did not alter the transcription neither the translation of both vectors. Consequently, we pursued the infection experiments on *DNAI1*-mutated HAECs with the Kozak modified/*DNAI1* conserved pair of vectors (with and without *HA* tag).

We were unable to detect endogenous DNAI1 protein and DNAI1 HA-tagged protein on Western blot of cultured normal HAECs presumably because the amount of extracted proteins from cultured HAECs was too low. However, translation from the various vectors was confirmed in transfected 293Bosc cells.

We also failed to label HA-tagged DNAI1 protein in the cilia of infected HAECs. However, the observation of the rescue of ciliary beating after *DNAI1* treatment of *DNAI1*-mutated HAECs with either one of the selected vectors supported the idea that normal DNAI1 proteins, HA-tagged or not, were efficiently localized to cilia. This functional evidence was reinforced by ultrastructural analysis showing an increased number of ODA in axonemes with tagged and untagged DNAI1 proteins. Beside these observations, no difference in the number of IDA was detected between control and *DNAI1*-treated HAECs, in agreement with the knowledge that DNAI1 proteins specifically belong to ODA.

In *DNAI1*-mutated HAECs treated by *DNAI1*, a partially uncoordinated ciliary beating was observed, probably because as we demonstrated in early experiments, about half of infected cells were not transduced. As a consequence, HAECs aggregates are mosaics composed of cells with immotile cilia and other cells with beating cilia. The number of ODA per axoneme in treated *DNAI1*-mutated HAECs is lower than normal because these values reflected a mix population of HAECs: efficiently transduced cells and untransduced cells, as shown in the bimodal distribution of ODA per axoneme in Figure 6. In this respect, our results are consistent with the recessive inheritance type of PCD disease for which a normal copy of the *DNAI1* gene is sufficient to an absence of symptoms.

In order to improve the number of rescued HAECs, we need to improve transduction efficiency, but respiratory epithelium is not exclusively composed of ciliated cells and at least seven other cell types are present [30]. To produce viral particles which would preferentially target human airway ciliated cells, the VSV-G large cell tropism protein could be replaced by another envelope protein from one of the various viruses known to infect airway epithelium, ie. coronavirus or Influenza virus [31],[32]. Influenza virus receptors, as α2,3-linked sialic acid receptors, were described to be specifically localized on ciliated airway and type II alveolar epithelial cells in both mouse and human respiratory epithelium, and on a subpopulation of basal cells in humans [32]. By contrast, α2,6-linked sialic acid receptors are expressed on human ciliated and goblet cells but not in the mouse. Therefore, it should be interesting to evaluate on our current culture model, the use of hemagglutinin (HA) from selected Influenza viruses which preferentially bind to ciliated cells. Finally, Szecsi *et al.* demonstrated that HA envelope protein of H7N1 and H5N1 avian viruses was successfully associated with retroviral-based vectors resulting in higher titres of infected cells than with VSV-G [33].

Airway ciliated cells are differentiated cells which do not proliferate and their cycle life span is supposed to be relatively short. In the mouse model, ciliated cells renewal was reported to be provided by Clara cells which are also essential in the alveolar type II cells generation [34]. Nevertheless, Park *et al*. reported that after an injury event, murine ciliated epithelial cells could transdifferentiate into other cell types and restored the airway epithelium [35]. In the human airway epithelium, Hajj *et al*. demonstrated that basal cells were able to regenerate as well as to differentiate into secretory and ciliated cells [36]. Targeting these basal cells with a lentiviral vector would result in the stable integration of the therapeutic gene with no need to repeat infections. The specificity of cells' targeting has been recently improved by taking into account the presence of proteases at the cell surface [37]. Thus, cell transduction can be achieved only in cell types that have the capacity to bind viral surface glycoprotein and to cleave the protease substrate because the surface glycoprotein of several enveloped viruses are expressed as precursor proteins that need to be cleaved into two subunits by cellular proteases to promote cell entry.

Finally, *DNAI1* gene transfer could be applied to other genes causing PCD, as *DNAH5* mutations are responsible for at least 28% of PCD cases [21]. However, another vector should be considered for this gene because of size limitation of lentivirus. As an alternative, AAV vectors offer a good safety profile in particular because viral DNA does not integrate into the genome of transfected cells. However, AAV repeated administration triggers anti-viral immune response and its packaging capacity is too limited to hold the *DNAH5* gene (13.9 kb). High-capacity episomal vectors offer the possibility to deliver not only a large gene but also the whole genomic locus including regulatory regions which ensures a physiological expression with tissue and time specificity. However, delivery of extrachromosomal DNA with non viral or viral strategies is so far limited to cell lines. This system, tough, is appropriate for long-term treatment as in PCD because chromosome-based episomal systems contain elements enabling the cell to process episome as an additional chromosome with a mitotic stability >99% in cell lines [38].

## Material and Methods

### Patient Data

The diagnosis of a male patient affected by PCD was based on clinical signs (recurrent upper and lower respiratory tract infections since early in life, nasal polyps and complete *situs inversus* – Kartagener syndrome). In addition, he was sterile. Electron microscopy of the cilia showed no ODA. *Ex vivo* culture of epithelial airway cells according to Jorissen's method demonstrated that his cilia were 6 µm-long and totally immotile. Analysis of his DNA demonstrated that he harbored heterozygous compound *DNAI1* mutations: c.48+2_48+3insT and c.1543G>A [20]. This patient signed an informed consent allowing us to experiment on nasal biopsies which consisted in removing the most anterior part of his middle nasal turbinate on both side. This study complies with the rules of the local ethical committee.

### Human Airway Epithelial Cell Culture

Human airway epithelial cells (HAECs) from normal subjects were obtained from nasal turbinates which were removed and discarded in the process giving access to the ethmoidal sinus. Patients were operated for tumours located in the ethmoidal region and had no respiratory disease.

Cells from control subjects and the patient were grown using the immerged cell culture previously described by Jorissen *et al*. [27]. Briefly, ciliated cells were isolated and cultured the day following the biopsy (J+1) in collagen-coated flasks to de-differentiate in non-ciliated cells. When they reached 80–90% confluence, collagen was digested (J', 7–10 days post-seeding) and cells were suspended in flasks with rotation to re-differentiate in the form of ciliated vesicles.

Cells were infected at J+1 or J+3. Non-ciliated cells were harvested at J+7 to 10 (J') and re-ciliated cells were fully re-differentiated at J'+28.

### Cloning of *DNAI1* cDNA

To generate *DNAI1* cDNA (AF091619), we extracted total RNA from ciliated HAEC and synthesized the cDNA. A pair of primers was designed to amplify the full-length cDNA. Six supplementary sets of specific primers were used to sequence *DNAI1* cDNA. The full-length *DNAI1* cDNA PCR product was cloned.

Addition of enzyme restriction sites in 5′ and 3′ *DNAI1* cDNA sequence was performed using two sets of primers. The BAMHIDNAI1_for and NCOIDNAI1_for primers (forward) added *Bam*H I and *Nco* I restriction sites upstream *DNAI1* cDNA sequence, respectively. The XHOIDNAI1_rev primer (reverse) added a *Xho* I restriction site downstream *DNAI1* cDNA sequence.

Addition of hemagglutinin (*HA*) tag downstream *DNAI1* cDNA sequence was performed by PCR using lentiviral vectors containing *DNAI1* cDNA (pK+*DNAI1* or pK-*DNAI1*) as template and upDNAI1_for (forward)/lowHA_rev (reverse) primers. For more information see Text S1.

### Lentiviral Vectors

The lentiviral vector system used in this study was derived from SIV vectors described by Negre *et al*. [25],[26]. Five different lentiviral vector constructs were used all under the transcriptional control of the human Elongation Factor-1 promoter (EFS): (1) pR4SA-EFS-GFP-W (pGFP), (2) pK-*DNAI1*, (3) pK+*DNAI1*, (4) pK-*HA*, (5) pK+*HA*. The pGFP vector contains the *eGFP* cDNA sequence. The pK-*DNAI1* and pK+*DNAI1* vector constructs are essentially the same as the pGFP vector but with the *DNAI1* cDNA sequence replacing the *eGFP* gene sequence, using *Bam*H I/*Xho* I and *Nco* I/*Xho* I sites, respectively (Figure 2A). The pK-*DNAI1* vector has a modified Kozak sequence with an intact *DNAI1* cDNA sequence whereas pK+*DNAI1* has an intact Kozak sequence with a modification at position 4 of the *DNAI1* cDNA sequence, resulting in DNAI1 second amino acid modification: isoleucine>valine. The pK-*HA* and pK+*HA* vector constructs correspond to pK-*DNAI1* and pK+*DNAI1* vectors, respectively, with a 3′ *DNAI1* cDNA hemagglutinin (*HA*) tag addition, using *Blp* I/*Xho* I restriction sites (Figure 2B).

### Lentiviral Transduction

Three sets of experiments were carried out.

In the first set, normal HAECs were transduced the day of seeding (J+1) on collagen-coated flasks or at J+3, and the cells were incubated for 24 hours with the lentivirus at MOI (Multiplicity Of Infection) of 7, 35 and 75 in the presence or absence of 6 µg/mL Polybrene (1,5-dimethyl-1,5-diazaundecamethylene polymethobromide, hexadimethrine bromide). MOI is the ratio of infectious agents (e. g. lentivirus) to infection targets (e. g. cells). Then, medium was completely changed in order to remove debris and inactive lentiviruses. Two days post-infection, reporter gene expression was analyzed by FACS.

In the second set of experiments, normal HAECs were transduced or not the day of seeding (J+1) at MOI75 with Polybrene with pK-*HA* or pK+*HA*. RNA was extracted before or after re-ciliation.

In the third set of experiments, transduction was performed on *DNAI1*-mutated HAECs at J+1, at MOI 75, with the use of Polybrene and cells were cultured to generate ciliated vesicles.

### RT–PCR Analysis

Tagged *DNAI1* gene expression was revealed by reverse transcription PCR (RT-PCR) on infected HAEC. Non-ciliated cells were collected the day of collagen digestion (J') and ciliated cells were collected when they were fully covered by cilia (J'+28).

Poly(A)^+^ mRNA was isolated by the Dynabeads Oligo(dT)_25_ purification kit, according to the manufacturer's protocol (Dynal Biotech, Norway).

### Western Blotting Analysis

Transient transfection of 293Bosc cells was performed with each four different plasmids: pK-*DNAI1*, pK+*DNAI1*, pK-*HA* or pK+*HA*. Western blot analysis was carried out according to standard techniques (Text S1).

### Video Analysis and Ciliary Beat Frequency (CBF)

In order to assess the functional activity of the ciliated *DNAI1*-mutated cells after lentiviral transduction, video recordings were performed with a ×40 objective lens in the light path at different steps of the culture in flasks, by using an Olympus IX50 inverted phase-contrast microscope. The control eGFP fluorescence was observed with a magnifying digital camera SCION CFW 1308M (Scion Corporation, Frederick, MD) and the recovery of ciliary beating was recorded with high speed digital video camera pco.1200 hs (PCO, Germany). The digital image-sampling rate was software-controllable using CamWare and for all experiments the sampling rate was set at 500 frames per second (fps).

For measurements of ciliary beat frequency (CBF), the video images of active ciliated cells were captured with a ×100 oil-immerged objective lens, using Leica DMRXA microscope and pco.1200 hs camera. A time-motion representation was obtained and analyzed using ImageJ software (National Institutes of Health, USA). Briefly, a line cutting cilia close to their tip was drawn. The “reslice” function of ImageJ was then used to obtain the video signal along this line (y axis) during 400 msec (x axis). Finally, the data were expressed as mean±SD from at least three regions of interest. Values for normal HAECs were used as controls.

### Transmission Electron Microscopy (TEM) and Statistical Analysis

Transmission electron microscopic analyses were processed on ciliated vesicles obtained after HAECs culture, as described by Jorissen *et al*. [27]. Amounts of ODA were expressed as mean±SD from n axonemes (n = 17 for pGFP, n = 21 for pK-*DNAI1*, n = 11 for pK-*HA*). Values of HAEC infected with pGFP, pK-*DNAI1* or pK-*HA* were statistically analysed by Student t test and the significance was calculated for a two-tailed test.

See Text S1 for more details.

## Supporting Information

Text S1 Supporting Materials and Methods.
(0.10 MB PDF)

Figure S1 HA-tagged *DNAI1* expression in 293Bosc cell line transfected with pK-*HA* vector. (A) Nuclei were labelled with DAPI (blue) and image was superimposed with transmission image. (B) HA tag was immunostained using anti-HA antibody associated with biotinylated goat anti-mouse and FITC-conjugated ExtrAvidin (green). A specific cytoplasmic localization was observed. Scale bars, 10 µm.
(2.21 MB TIF)

Figure S2 *DNAI1*-mutated HAEC transduced with pGFP vector. eGFP fluorescence superimposed with transmission image. (A) At J+3, HAEC are de-differentiated and adherent (bar, 50 µm). (B) At J'+17, HAEC are re-differentiated and in suspension as ciliated vesicles. Arrowheads indicate cilia (bar, 20 µm).
(2.54 MB TIF)

Video S1 *DNAI1*-deficient Human Airway Epithelial Cells transduced with GFP. *DNAI1*-mutated Human Airway Epithelial Cells were transduced with a GFP containing lentiviral vector. Cilia are completely immotile as in non-transduced cells of this patient. The video was recorded at 500 frames per second. This video includes 201 frames.
(7.23 MB AVI)

Video S2 *DNAI1*-mutated Human Airway Epithelial Cells after transduction with *DNAI1* lentiviral vectors. *DNAI1*-mutated Human Airway Epithelial Cells were transduced with a pK-*DNAI1* and pK-*HA* lentiviral vectors. In both cases, beating cilia were clearly visible as on this video from pK-*DNAI1* treated cells. The video was recorded at 500 frames per second. This video includes 200 frames.
(7.19 MB AVI)
