# Supplementary material for: Ciliary Beating Recovery in Deficient Human Airway Epithelial Cells after Lentivirus Ex Vivo Gene Therapy
Source: PLoS Genet. 2009 Mar 20;5(3):e1000422. doi: 10.1371/journal.pgen.1000422 (PMC2650261; doi:10.1371/journal.pgen.1000422)
Supplement: Text S1 — Supporting Materials and Methods. (0.10 MB PDF) [file pgen.1000422.s001.pdf]

SUPPORTING INFORMATION

MATERIAL and METHODS

Human Airway Epithelial Cell Culture

Human airway epithelial cells (HAECs) from normal subjects were obtained from nasal turbinates which were removed and discarded in the process giving access to the ethmoidal sinus (from the department of Ear, Nose and Throat, Hôpital de la Croix-Rousse, Lyon, France). Patients were operated for tumours located in the ethmoidal region and had no respiratory disease.

Cells from control subjects and the patient were grown using the immerged cell culture previously described by Jorissen *et al.* (1). Briefly, ciliated cells were isolated by pronase digestion overnight and expanded the day following the biopsy (J+1) in collagen-coated 25 cm^2 flasks (10-15,000 cells/cm^2) to de-differentiate in non-ciliated cells at 37°C, 5% CO_2. When they reached 80-90% confluence, collagen was digested (J', 7-10 days post-seeding) and cells were suspended in flasks with rotation (inclination 10°, 80 rpm) at 37°C, to re-differentiate in the form of ciliated vesicles.

Cells were infected at J+1 or J+3. Non-ciliated cells were harvested at J+7 to 10 (J') and re-ciliated cells were fully re-differentiated at J'+28.

Cloning of *DNAI1* cDNA

To generate *DNAI1* cDNA (AF091619), we extracted total RNA from ciliated HAECs with Extract-All (Eurobio, France), following manufacturer's instructions. cDNA was synthesized by using P5 specific primer (reverse: 5'-AAGGAACAGAACAAGAAGGGAA-3') and

SuperScript II Reverse Transcriptase, as recommended by the manufacturer (Invitrogen, Grand Island, NY). The P4 (forward: 5'-TTGTGTGTGGTCAAGGAGACGG-3') and P5 specific primers were designed to amplify the full-length double-stranded cDNA (2319 bp) with the Expand High Fidelity PCR System kit, according to manufacturer's instructions (Roche Applied Science, France). Six Supplementary sets of specific primers were used to amplify (products from 371 to 594 bp) and to sequence *DNAI1* cDNA (primer sequences available upon request). Purified PCR products were sequenced using the Big Dye® sequencing chemistry (Applied Biosystems, Foster City, CA). Full-length *DNAI1* cDNA PCR product was cloned into pCR®II-TOPO® vector and used to transform DH5α One Shot competent cells, according to the manufacturer's protocol (Invitrogen). Cloned products were sequenced as previously described.

Addition of enzyme restriction sites in 5' and 3' *DNAI1* cDNA sequence was performed using two sets of primers. The BAMHIDNAI1_for and NCOIDNAI1_for primers (forward) added *Bam*H I and *Nco* I restriction sites upstream *DNAI1* cDNA sequence, respectively. The XHOIDNAI1_rev primer (reverse) added a *Xho* I restriction site downstream *DNAI1* cDNA sequence.

Addition of hemagglutinin (*HA*) tag downstream *DNAI1* cDNA sequence was performed by PCR using lentiviral vectors containing *DNAI1* cDNA (pK+*DNAI1* or pK-*DNAI1*) as template and upDNAI1_for (forward)/lowHA_rev (reverse) primers with the Expand High Fidelity PCR System kit, following manufacturer's instructions (Roche Applied Science).

RT-PCR Analysis

Tagged *DNAI1* gene expression was revealed by reverse transcription PCR (RT-PCR) on infected HAECs. Non-ciliated cells were collected the day of collagen digestion (J') and ciliated cells were collected when they were fully covered by cilia (J'+28).

Poly(A)$^+$ mRNA was isolated by the Dynabeads Oligo(dT)$_{25}$ purification kit, according to the manufacturer's protocol (Dynal Biotech, Norway). cDNA was synthesized by tag specific priming, using HART_rev (reverse: 5'-GGCATAGTCGGGGACGTCGTA-3') or P5 *DNAI1* specific primer, and SuperScript II Reverse Transcriptase, following manufacturer's instructions (Invitrogen). A specific pair of primers located on *DNAI1* cDNA sequence (DNAI1seq5.2F/DNAI1seq5.2R, located in exons 13-14 and exon 19, respectively) was used to amplify a 545 bp PCR product under standard conditions.

Absence of genomic DNA contamination was confirmed by PCR with *alpha-tubulin* primers which could amplify either a 320 bp fragment on cDNA or a 468 bp fragment on genomic DNA (protocol is available upon request).

<u>Viral Vector Production and Titration</u>

The lentiviral vector was produced and titrated as described by Negre *et al.* (2, 3). The lentiviral vector was produced by transient transfection of 293T cells (100 mm dish) with three different plasmids, ie. 9 µg of the vector construct, 8 µg of SIV helper construct (pSIV3+) and 4 µg of VSV-G encoding plasmid (pG-REV), using calcium phosphate co-precipitation, as described by Negre *et al.* (2, 3).

For larger stocks, the procedure was scaled up to 10-15 dishes and the supernatants harvested at 24 and 36 hours post-transfection before clarification, filtration using a 0,45 µm filter, and concentration in SW28 (25,000 rpm, 2 hours and 30 minutes). Viruses were resuspended in 1X PBS containing 1% glycerol for 2 hours on ice, pooled, aliquoted and stored at -80°C.

Titration was performed using serial dilutions (volumes: 1, 0.1 and 0.02 µL) of the pGFP vector stock to transduce 2×10^6 sMAGI cells in the presence of Polybrene (6 µg/ml; Sigma-Aldrich, Saint Louis, MO). FACS analysis 48 hours post-transduction gave the proportion of

GFP-positive cells and allowed us to calculate the amount of particles inoculated at day 0. Titres of $\geq 10^9$ IU/ml were obtained (IU, infectious units).

Assay for detection of replication competent virus

To detect presence of replication competent virus (RCR) in viral preparations, we followed the French Commission de Génie Génétique's recommendations (notice 4). Briefly, to be downgraded in category 2 laboratories, lentiviruses which were produced in category 3 laboratories had to be controlled: after permissive cells transduction, transduced cells supernatant was analyzed 7 days post-infection. The samples were assayed for SIV P27 capsid protein by ELISA and undetectable signals versus a control of non-transduced cells confirmed absence of RCR.

Western Blotting Analysis

Total proteins lysates from transfected 293Bosc cells were analyzed for DNAI1 and HA-tagged DNAI1 proteins expression. Non-transfected 293Bosc cells do not express DNAI1 proteins.

DNAI1 and HA-tagged DNAI1 proteins were produced by transient transfection of 293Bosc cells (35 mm dish) with each four different plasmids: pK-*DNAI1*, pK+*DNAI1*, pK-*HA* or pK+*HA*.

About 2.5×10^5 cells were plated one day before transfection. The day of transfection, 1 µg of each plasmid was diluted in 25 µL 150 mM NaCl and 5 µL of ExGen500 (Euromedex, France) was added to 20 µL of 150 Mm NaCl. This last mix was added to diluted plasmids, incubated ten minutes at room temperature before transfer on 293Bosc cells. Cells were incubated for three hours with plasmids at 37°C, 5% CO_2, and then supplementary medium was added for 12-hours incubation before complete changing. Forty-eight hours post-transfection, medium was removed; cells were rinsed with cooled 1X PBS, detached from the

dish using a scraper and centrifuged at 70 g for 5 minutes. The supernatant was removed and the pellet was stored at -80°C or was resuspended with an extraction buffer (1X TE: 25 mM Tris, 2 mM EDTA, 0.5 mM DTT, pH 7.75, 0.1% Tween-20) containing protease inhibitor cocktail (PIC, P8340, Sigma-Aldrich), according to the manufacturer's instructions.

Total protein lysates were analyzed on 8% SDS-polyacrylamide gels (10 µg per lane). After proteins electrophoretic transfer to Hybond-P PVDF membrane (GE Healthcare Life Sciences, Germany), non-specific sites were blocked for 1 hour at room temperature in TBST containing 5% non-fat milk (TBST: 1% Tween-20 in Tris-Buffer Saline, 100 mM Tris, 1.5 M NaCl, 7.8<pH<8.2). Membranes were blotted with anti-dyn69 polyclonal rabbit antibody, 1/1000 (anti-DNAI1 raised against the peptide NNPVRDRECQTEPPPR, Eurogentec), anti-HA monoclonal mouse antibody, 1/4000 (H3663, Sigma-Aldrich), or anti-actin polyclonal rabbit antibody, 1/4000 (A2066, Sigma-Aldrich). As secondary antibody, a peroxidase-conjugated anti-rabbit antibody (1:10,000 dilution; NA9340V, GE Healthcare Life Sciences) or a peroxidase-conjugated anti-mouse antibody (1:25,000 dilution; NA931, GE Healthcare Life Sciences) were used depending on the first antibody. Proteins were detected by chemiluminescence using ECL Plus Western Blotting Detection Reagents (GE Healthcare Life Sciences). The specificity of anti-dyn69 polyclonal antibody was demonstrated by Western blot using ciliated and non-ciliated tissues, a band of about 80 kD appearing only on ciliated tissues.

Transmission Electron Microscopy (TEM) and statistical analysis

At day J'+31 (31-days post-collagen-digestion), ciliated cells were fixed in 3% glutaraldehyde in 0,1 M sodium cacodylate buffer (pH 7.4) for two to three hours, followed by an additional OsO_4 0,1 M phosphate buffer (pH 7.4) fixation for one hour. The material was dehydrated in a

graded ethanol series and then embedded in epoxy resin. One micron sections were prepared for TEM and axoneme ultrastructure analysis.

REFERENCES

1. Jorissen M, Willems T, Van der Schueren B, Verbeken E, De Boeck K. Ultrastructural expression of primary ciliary dyskinesia after ciliogenesis in culture. *Acta Otorhinolaryngol Belg* 2000;54:343-356.

2. Negre D, Mangeot PE, Duisit G, Blanchard S, Vidalain PO, Leissner P, Winter AJ, Rabourdin-Combe C, Mehtali M, Moullier P, Darlix JL, Cosset FL. Characterization of novel safe lentiviral vectors derived from simian immunodeficiency virus (sivmac251) that efficiently transduce mature human dendritic cells. *Gene Ther* 2000;7:1613-1623.

3. Negre D, Duisit G, Mangeot PE, Moullier P, Darlix JL, Cosset FL. Lentiviral vectors derived from simian immunodeficiency virus. *Current topics in microbiology and immunology* 2002;261:53-74.
