# Supplementary figures and images for: Ciliary Beating Recovery in Deficient Human Airway Epithelial Cells after Lentivirus Ex Vivo Gene Therapy
Source: PLoS Genet. 2009 Mar 20;5(3):e1000422. doi: 10.1371/journal.pgen.1000422 (PMC2650261; doi:10.1371/journal.pgen.1000422)

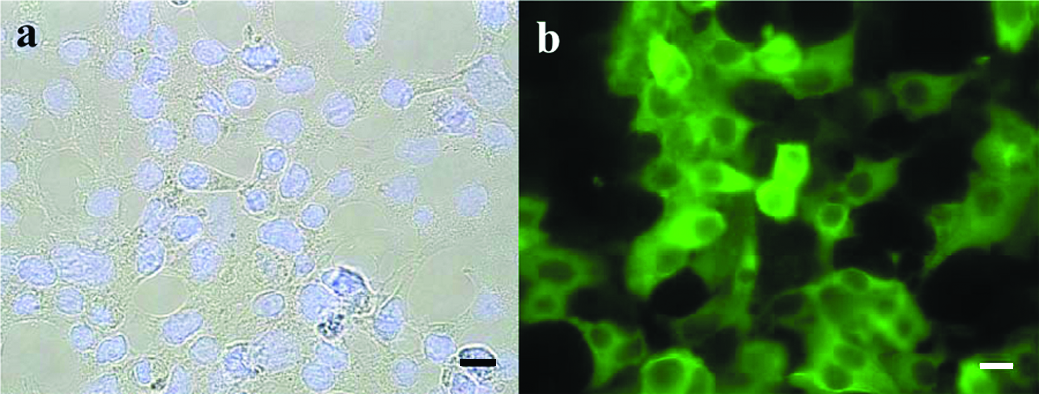

Supplement: Figure S1 — HA-tagged DNAI1 expression in 293Bosc cell line transfected with pK-HA vector. (A) Nuclei were labelled with DAPI (blue) and image was superimposed with transmission image. (B) HA tag was immunostained using anti-HA antibody associated with biotinylated goat anti-mouse and FITC-conjugated ExtrAvidin (green). A specific cytoplasmic localization was observed. Scale bars, 10 µm. (2.21 MB TIF) [file pgen.1000422.s002.tif]

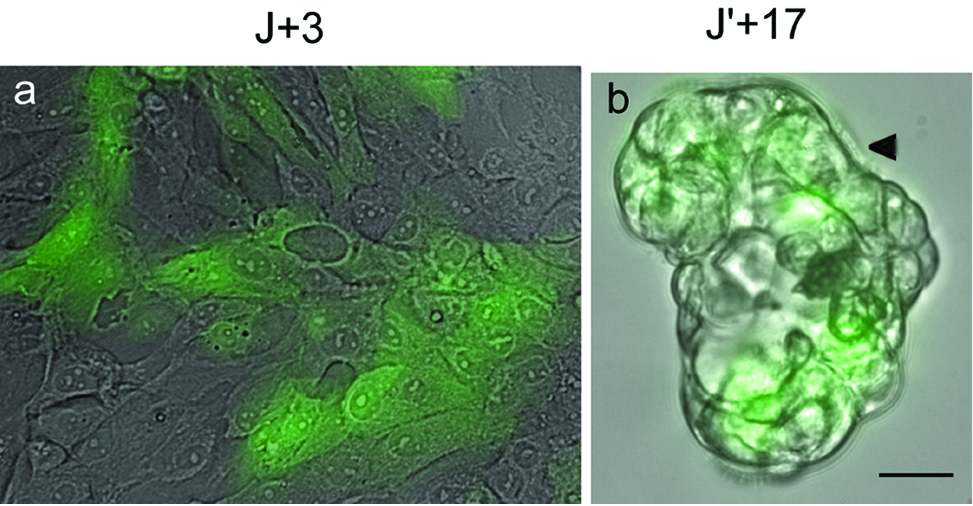

Supplement: Figure S2 — DNAI1-mutated HAEC transduced with pGFP vector. eGFP fluorescence superimposed with transmission image. (A) At J+3, HAEC are de-differentiated and adherent (bar, 50 µm). (B) At J'+17, HAEC are re-differentiated and in suspension as ciliated vesicles. Arrowheads indicate cilia (bar, 20 µm). (2.54 MB TIF) [file pgen.1000422.s003.tif]
